# Bedrock erosion in subglacial channels

**DOI:** 10.1371/journal.pone.0253768

**Published:** 2021-09-09

**Authors:** Sergio Fagherazzi, Luca Baticci, Christine M. Brandon, Maria Cristina Rulli

**Affiliations:** 1 Department of Earth & Environment, Boston University, Boston, Massachusetts, United States of America; 2 Department of Hydraulic, Roadways, Environmental, and Surveying Engineering Politecnico di Milano, Milan, Italy; 3 Science and Mathematics Center, Bridgewater State University, Bridgewater, Massachusetts, United States of America; Duy Tan University, VIETNAM

## Abstract

The Labyrinth in the McMurdo Dry Valleys of Antarctica is characterized by large bedrock channels emerging from beneath the margin of Wright Upper Glacier. To study the morphodynamics of large subglacial channels cut into bedrock, we develop herein a numerical model based on the classical theory of subglacial channels and recent results on bedrock abrasion by saltating bed load. Model results show that bedrock abrasion in subglacial channels with pressurized flow reaches a maximum at an intermediate distance up-ice from the glacier snout for a wide range of sediment grain sizes and sediment loads. Close to the snout, the velocity is too low and the sediment particles cannot be mobilized. Far from the snout, the flow accelerates and sediment is transported in suspension, thus limiting particle impacts at the channel bottom and reducing abrasion. This non-monotonic relationship between subglacial flow and bedrock abrasion produces concave up bottom profiles in subglacial channels and potential cross-section constrictions after channel confluences. Both landforms are present in the bedrock channels of the Labyrinth. We therefore conclude that these geomorphic features are a possible signature of bedrock abrasion, rather than glacial scour, and reflect the complex interplay between transport rate, sediment load, and transport capacity in subglacial channels.

## 1. Introduction

The hydrodynamics of glacio-fluvial systems have been studied for many decades. In particular, much work has been done to quantify subglacial pressure distribution [1–4], leading to a description of subglacial drainage networks [5–7]. Several of these results have been verified by field evidence (e.g. [8]) and different categorization schemes for subglacial ice conduit geometries have also been proposed [4].

Subglacial channels can be cut upward into ice (R-channels, [1]) or incised into bedrock (N-channels, [9]). R-channels form because of a balance between channel enlargement by ice melting due to energy dissipation of water flow and closure by ice creeping in the channel [1]. N-channels imply a rate of bedrock erosion by water flow higher than the basal glacier erosion, so that the mix of water and sediments below a glacier is able to carve a channel in the bedrock [10]. In R-channels the water pressure decreases as a function of water discharge, so that ultimately a few large conduits, tens of meters wide, grow at the expense of smaller conduits by collecting melt water that naturally moves towards areas of low pressure.

All water flow beneath glaciers is not confined in conduits (e.g. R- and N-channels), but instead is often distributed across a drainage system of cavities at the glacier bed between the ice and bedrock [11, 12] showed that a system of cavities linked by small orifices stems from the ice-bedrock separation due to basal sliding. In this drainage system, the water flux increases as a function of water pressure, since higher pressures are required to drive the flow through the orifices (see also [13]). Moreover, the resulting higher pressures facilitate the sliding of the glacier and the formation of new cavities. As a result, a dendritic network of cavities with dimensions of only a few meters forms, since the enlargement of a single cavity is hampered by the increasing pressure necessary to drive the flow [12]. Subglacial flow can also concentrate in canals cut into subglacial sediments, when a thick layer of till is present below the glacier [5].

While many of these studies reveal the final arrangement of subglacial channels, cavities, and canals in drainage networks, few of them consider the processes responsible for bedrock incision. Bedrock erosion can occur by abrasion, plucking, or cavitation (e.g. [14]). Abrasion was likely dominant in the Labyrinth because the dolerite is massive and coarse-grained, so tends to erode to produce course sands rather than fracture into blocks.

More work on this topic comes from studies of bedrock rivers [15–20]. Models and experiments from these authors relate the incision and evolution of bedrock channels to sediment abrasion. A key result is a non-linear dependence of bedrock abrasion on sediment supply and transport rate, with the maximum wear occurring for intermediate values of these parameters. A low sediment load does not provide enough abrasion tools to scour the bedrock, whereas a high sediment load yields alluviation, with deposited sediments protecting the bedrock from erosion. This process is known as the tools and cover effect [16].

Bottom shear stress is also a main driver of bedrock erosion. In both the models of [17] and [18], abrasion is due to the impact of saltating sediments. If the flow velocity and related bottom shear stress are too low (i.e. low transport capacity), sediment is unable to be mobilized and therefore abrasion is negligible. On the other hand, when the flow is very fast and the transport capacity is high, all the sediment is transported in suspension and abrasion at the bed is again very limited since the frequency of impacts with the bed is low. Again, as in the case of sediment supply, the maximum erosion rate occurs for intermediate values of the transport rate [17]. The presence of optimal conditions for abrasion indicate that bedrock conduits under glaciers might develop for a specific range of hydrodynamics and sedimentological parameters, whereas abrasion is hampered for very low flows and sediment supplies and for very fast flows and large sediment supplies.

This paper focuses on the morphodynamic consequences of a non-monotonic relationship between erosion rates and sediment supply in subglacial channels incised in bedrock. Model results are compared to the morphology of subglacial bedrock channels in the Labyrinth, Antarctica. The model is used to determine what factors are important in influencing bedrock abrasion in a subglacial environment and how these affect erosion along the entire length of a subglacial channel.

## 2. Geologic setting of the Labyrinth

**Fig 1:**
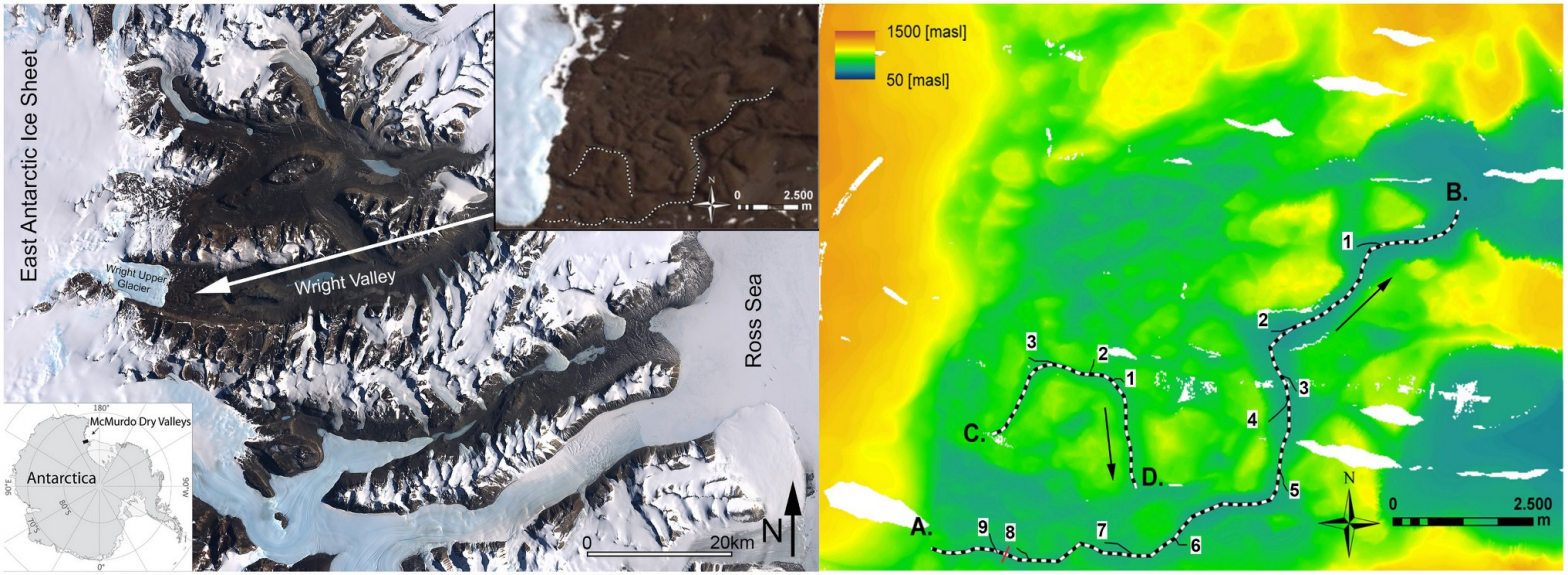
Left: Satellite image of the Dry Valleys region derived from the Landsat Image Mosaic of Antarctica (LIMA). The Labyrinth is indicated with a white arrow. Inset at bottom left shows location of the Dry Valleys in Antarctica. Inset at the top right shows a close up of the Labyrinth with the two channels presented in this study highlighted in white. Right: Digital Elevation Model extracted from LiDAR data showing the axis of two subglacial cannels (channel A-B and C-D). Confluences of smaller channels into the two channels are indicated with numbers. In red the cross section used in the model. The black arrows indicate the flow direction.

The McMurdo Dry Valleys (also known as the Dry Valleys), encompass one of the few ice-free areas in Antarctica. These valleys, which lie in the Transantarctic Mountains between the East Antarctic Polar Plateau and seasonally open waters of the Ross Sea (Fig 1), include the bedrock channels of upper Wright Valley, called the Labyrinth. The Labyrinth emerges from beneath the margin of Wright Upper Glacier and extends ~10 km to the east (Fig 1). Where fully exposed, it is ~7 km wide, occupies an elevation range from ~850 m to ~1200 m, and is incised into a generally flat-lying (~3° W dip) sill of Ferrar Dolerite. [21] identified anastomosing, U-shaped channels incised in bedrock occurring below 850 m elevation. The largest channels are up to 600 meters wide and 250 meters deep. Several geomorphic features, including extreme reverse gradients along longitudinal channel profiles (with relief as high as 80 m) and potholes up to 30 meters in diameter at tributary junctions [21–23] suggest that the channels formed by erosion due to large-scale subglacial floods, rather than from typical basal glacier erosion.

Based on the size of blocks transported by the subglacial flood and the cross-sectional area of the channels [21], estimated a maximum discharge of 1.6–2.2x10^6^ m^3^/s. Large scale numerical simulations of subglacial flow carried out by [24] indicate that such large discharges are only possible during outburst floods from subglacial lakes. These subglacial drainage events are episodic, and occur when meltwater trapped in upstream subglacial lakes is released [24]. Radiometric dates on reworked and in-situ ashfall found in this area suggest that the last major subglacial flood in the Labyrinth occurred during the middle Miocene climate transition between 12.4 and 14.4 Mya [23, 25–28]. During this time the Antarctic ice sheet expanded and changed from a dynamic, wet-based ice sheet to its current cold-based state, reducing meltwater runoff. Since then, the area has undergone very little morphologic change, with cosmogenic-nuclide surface-exposure-age analyses indicating a rate of bedrock erosion of ~5–50 cm Ma^-1^ [29–31].

## 3. Methods

### 3.1 Water pressure in subglacial channels

**Fig 2:**
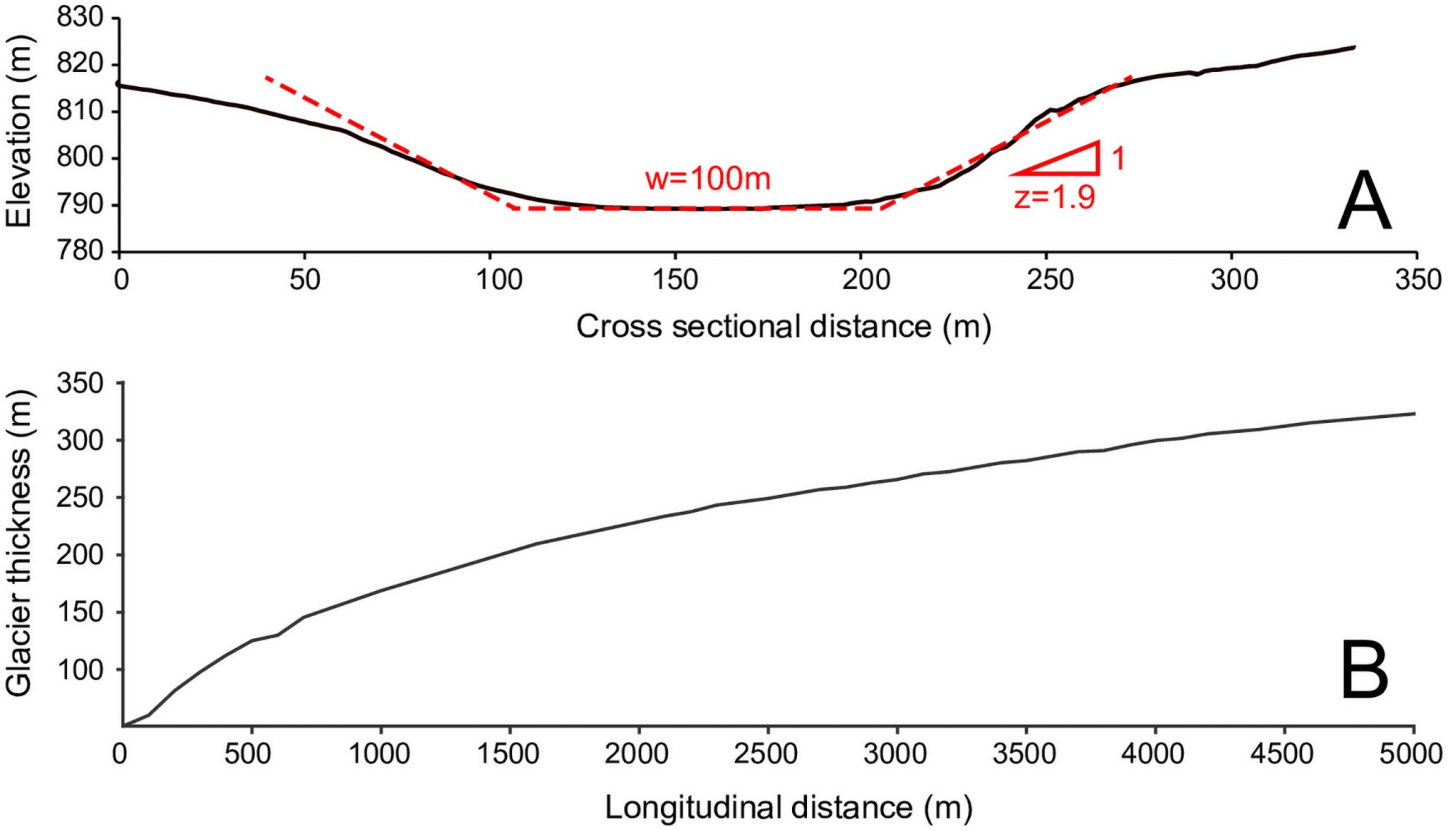
a) Typical cross section in the Labyrinth, Antarctica, derived from the LiDAR survey (black line) and the trapezoidal cross section adopted in the model (dotted red lines). The location of the cross section is indicated in red in Fig 1; b) glacier thickness profile reconstructed by Hall and Denton (2005) in the eastern Wright Valley, Antarctica.

**Fig 3:**
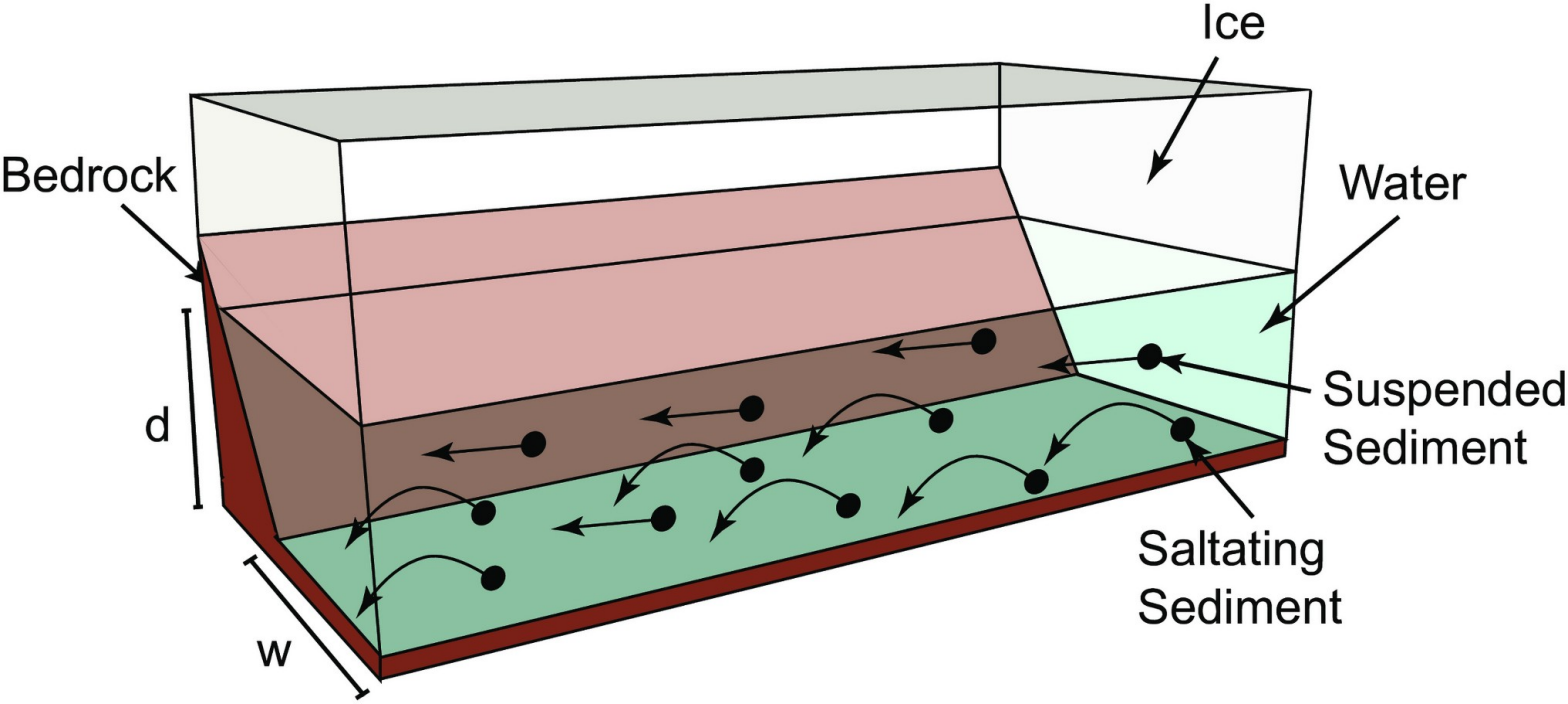
Illustration of a subglacial bedrock channel. Bedrock is present at three sides (brown; side closest to viewer not shown) and the glacier’s bottom provides an ice roof to the channel (white). Parameters shown are water depth, d, and channel width, w. The channel is completely filled with water (blue) that transports saltating (curved arrows) and suspended sediment (straight arrows).

Our model considers a trapezoidal channel that is dolerite bedrock on three sides and ice at the top. The channel has a fixed bottom width, *w*, set at 100 m, and a bank slope *z* of 1.9 m/m (Fig 2a), which is representative of channels in the Labyrinth, and a variable water depth, *d*. The width of the main channel is relatively constant (Fig 1), so to a first approximation we assume that the bottom width is constant, although the top width can increase with water depth. The water that flows through the channel is assumed to be at ice melting temperature (0°C). The basal ice of a glacier forms an ice roof over the subglacial bedrock channels (N-channel, Nye 1976). Because this ice roof can creep into or retreat from a subglacial channel, the depth of the channel occupied by flowing water is variable, depending on the speed of ice creep and the rate at which water melts the ice above. By assuming equilibrium between ice creep in the channel and melting of ice due to the water flow, a relationship between the water depth, *d*, the pressure, *p*, measured as piezometric head, and discharge, *Q* is derived. Once the pressure head is obtained, it is used to compute the bedrock erosion rate along the channel’s entire length, *L*. Fig 3 illustrates the geometric parameters of a subglacial channel.

The following derivation is based on the model outlined by [1], who was among the first to study water flow in a steady-state, subglacial system, but the equations have been modified herein to account for a bedrock channel. First, Manning’s equation for velocity, *v*, in the channel is defined as

$$v=\frac{1}{n}R_{h}^{2/3}S^{1/2}$$ (1)

where *n* is the Manning roughness coefficient, *R*_*h*_ is the hydraulic radius of the channel, and *S* is the energy slope. *R*_*h*_ is he ratio between cross-sectional area of the channel *A* and wet perimeter *P*. Substituting *v = Q/A* and computing the hydraulic radius as a function of channel width and depth for a channel filled with water, and expressing the gradient in pressure head as *S = dp/dx*, Eq 1 becomes:

$$\frac{dp}{dx}=\frac{n^{2}Q^{2}{\left(2\left(w+zd+d\sqrt{1+z^{2}}\right)\right)}^{4/3}}{{\left(d\left(w+zd\right)\right)}^{10/3}}$$ (2)

where *x* is the distance along the length of the channel from the glacier snout.

The total energy lost per unit length of channel per unit time is:

dE=Qdp (3)

However, not all of this energy is used to melt ice. Some, *dE*_*t*_, is used to raise the temperature of the water to the melting temperature which varies as a function of water pressure. The general expression for the energy used to melt ice, *dE*_*m*_, is therefore [1]:

$$dE_{m}=dE-dE_{t}$$ (4)

The energy loss by ice melting can be expressed as:

$$dE_{m}=c_{m}\rho_{i}dV_{m}$$ (5)

where *dV*_*m*_ is the volume of ice melted, *c*_*m*_ is the energy of fusion, *ρ*_*i*_ is the density of ice.

The energy necessary to raise the temperature of the water at high pressures is:

$$dE_{t}=c_{t}c_{w}\rho_{w}Qdp$$ (6)

where *c*_*t*_ is the change of pressure melting point with temperature, *c*_*w*_ is the specific heat capacity of water, and *ρ*_*w*_ is the density of water.

Substituting these expressions into (4) and solving for *dV*_*m*_, results in:

$$dV_{m}=\frac{Qdp}{c_{m}\rho_{i}}-\frac{c_{t}c_{w}\rho_{w}Qdp}{c_{m}\rho_{i}}$$ (7)

We can then impose that, at equilibrium, the volume of ice melted by the flowing water, *dV*_*m*_, should be equal to the volume of ice that creeps in the channel, *dV*_*c*_,. This volume is a function of the cross-sectional area, *A*, the effective pressure head *P—p* which is the difference between the ice overburden pressure *P* and the water pressure in the channel *p*, and ice flow parameters. From [1], the rate at which the channel is filled by ice creep is:

$$dV_{c}=d\left(w+zd\right){\left(\frac{P-p}{Bn_{1}}\right)}^{n_{1}}dx$$ (8)

where *B* and *n*_*1*_ are ice flow parameters (Table 1). This equation can now be set equal to Eq 7 and solved for *dp/dx*:

$$\frac{dp}{dx}=c_{m}\rho_{i}d\left(w+zd\right){\left(\frac{P-p}{Bn_{1}}\right)}^{n_{1}}{\left(Q-c_{t}c_{w}\rho_{w}Q\right)}^{-1}$$ (9)

**Table 1:** Constants.

Symbol	Name	Value (units)
B	Glen’s flow law parameter	7415.2 (m) [1]
C_1_	constant in Stoke’s eqn. for laminar settling	20 (unitless) [33]
C_2_	constant drag force on large diameter particles	1.1 (unitless) [33]
c_m_	energy of fusion	3.34x10^5^ (J kg^-1^) [1]
c_t_	change of pressure melting point	7.5x10^-8^ (deg J^-1^ m^3^)[1]
c_w_	specific heat capacity of water	4.22x10^3^ (J kg^-1^ deg^-1^)[1]
g	gravitational acceleration	9.81 (m s^-2^)
k_ν_	rock resistance coefficient	10^6^ (unitless) [17]
n	Manning’s roughness coefficient	0.05 (m^-1/3^ s) [1]
z	Bank slope	1.9 (m/m)
n_1_	Glen’s flow law parameter	3 (unitless) [1]
R	submerged specific gravity of a sediment grain	1.65 (unitless) [33]
Y	Young’s modulus of elasticity	5x10^10^ (Pa) [17]
ν_w_	kinematic viscosity of water	1.787x10^-6^ (m^2^ s^-1^) [17]
ρ_i_	density of ice	917 (kg m^-3^) [1]
ρ_s_	density of sediment grain	2910 (kg m-3) [15]
ρ_w_	density of water	999.84 (kg m^-3^) [1]
σ_t_	rock tensile strength	7x10^6^ (Pa) [17]
τ_c_	critical shear stress	0.03 (unitless) [17]

Source: Table 1. The first column contains the constants used in the equations in this paper, the second contains a brief description of them and the third contains their numerical values. Numbers in squared brackets in the third column refer to the papers from which these constants were obtained.

Finally, substituting (2) into (9) and solving algebraically, a non-linear expression for the water depth in the channel is derived:

$$d={\left[\frac{2.52n_{}^{2}Q^{3}{\left(w+zd+d\sqrt{1+z^{2}}\right)}^{\frac{4}{3}}g\rho_{w}}{{\left(w+zd\right)}^{\frac{13}{3}}c_{m}\rho_{i}}\;\left(1-c_{t}c_{w}\rho_{w}\right){\left(\frac{Bn_{1}}{P-p}\right)}^{n_{1}}\right]}^{\frac{3}{13}}$$ (10)

Eqs 2 and 10 must be solved together to determine the distribution of pressure head, *p*, and water depth, *d*, along a subglacial channel. Specifically, Eq 2 needs to be integrated to determine *p* along *x*; Eq 10 presents the unknown variable *d* on both sides, so it needs to be solved iteratively.

### 3.2 Bedrock erosion in subglacial channels

To compute bedrock erosion, the model of [17] for saltation-abrasion is modified by including parameters for pressurized water flow. This formulation accounts for both bedload and suspended load abrasion. The erosion rate, *ε*, is:

$$\begin{array}{cc} \varepsilon =\frac{0.08R_{b}gY}{k_{v}\sigma_{t}^{2}}\;q_{s}{\left(\frac{\tau^{*}}{\tau_{c}^{*}}-1\right)}^{-1/2}\;\left(1-\frac{q_{s}}{q_{t}}\right)\;{\left(1-{\left(\frac{u{*}_{}}{w_{f}}\right)}^{2}\right)}^{3/2} & \text{for}\;\tau^{*}>\tau_{c}^{*},\;q_{s}<q_{t}\\ \varepsilon =0\; & \text{for}\;\tau^{*}\leq \tau_{c}^{*},\;q_{s}\geq q_{t} \end{array}$$ (11)

where $R_{b}\;=\;\frac{\rho_{s}}{\rho_{w}}-1$ is the nondimensional buoyant sediment density, *ρ*_*s*_ is the density of the sediments, *ρ*_*w*_ is the density of water, *Y* is the Young’s modulus of elasticity of the bedrock, *k*_*ν*_ is the rock resistance coefficient, and *σ*_*t*_ is the rock tensile strength. The first term is a description of the bedrock’s resistance to erosion. The term $\frac{\tau^{*}}{\tau_{c}^{*}}\;$ is the ratio of the Shields parameter, $\tau^{*}\;=\;\frac{\tau_{b}}{\left(\rho_{s}-\rho_{w}\right)gD_{s}}$, above the nondimensional critical shear stress, *τ*_c_^*^, where *τ*_*b*_ is the bottom shear stress and *D*_*s*_ is the diameter of a sediment grain. The Shields parameter must be greater than *τ*_c_^*^ for erosion to occur. The term $\left(1-\frac{q_{s}}{q_{t}}\right)\;$ is the complement of the ratio of the supply of sediment per unit width, *q*_*s*_, to the sediment transport capacity per unit width, *q*_*t*_. In this term, *q*_*s*_ must be less than *q*_*t*_ or else the erosion rate will go to zero. The term ${\left(1-{\left(\frac{u*}{w_{f}}\right)}^{2}\right)}^{3/2}$ relates the flow shear velocity $u*=\sqrt{\frac{\tau_{b}}{\rho_{w}}}$ to the fall velocity of a sediment particle, *w*_*f*_, and dictates the propensity of particles to travel as suspended load or bedload.

The bottom shear stress can be related to the pressure head gradient with:

$$\tau_{b}=\frac{\rho_{w}gR_{h}}{L}\frac{dp}{dx}$$ (12)

while the transport capacity can be computed using the bedload sediment transport relation of [32] (see [17]):

$$q_{t}=5.7\rho_{s}{\left(R_{b}gD_{s}^{3}\right)}^{1/2}{\left(\tau^{*}-\tau_{c}^{*}\right)}^{3/2}$$ (13)

Furthermore the sediment fall velocity *w*_*f*_ is calculated as a function of grain size with the empirical expression put forward by [33]:

$$w_{f}=\frac{R_{b}gD_{s}^{2}}{18\upsilon +{\left(0.75R_{b}gD_{s}^{3}\right)}^{0.5}}$$ (14)

where *ν* is water viscosity.

### 3.3 Model formulation

A numerical scheme is used to calculate the erosion rate in a subglacial bedrock channel. We assume a total channel length of 5 km, and a critical Shields parameter ${\tau_{c}}^{*}\;=\;0.03$. We start at a location *i* where the pressure head *p*(*i*) is known. First, Eq 10 is solved using an iterative method to find the water depth at location *i*. As a first approximation, it is assumed that *d* << *w*, giving:

$$d\left(i\right)={\left[\frac{2.52n^{2}Q^{3}g\rho_{w}}{w^{3}c_{m}\rho_{i}}\;\left(1-c_{t}c_{w}\rho_{w}\right){\left(\frac{Bn_{1}}{P-p\left(i\right)}\right)}^{n_{1}}\right]}^{\frac{3}{13}}$$ (15)

This depth value *d*(*i*) is then substituted on the right hand side of Eq 10, to obtain a better approximation *d*(*i*)^*new*^:

$$d{\left(i\right)}^{new}={\left[\frac{2.52n_{}^{2}Q^{3}{\left(w+zd\left(i\right)+d\left(i\right)\sqrt{1+z^{2}}\right)}^{\frac{4}{3}}g\rho_{w}}{{\left(w+zd\left(i\right)\right)}^{\frac{13}{3}}c_{m}\rho_{i}}\;\left(1-c_{t}c_{w}\rho_{w}\right){\left(\frac{Bn_{1}}{P-p\left(i\right)}\right)}^{n_{1}}\right]}^{\frac{3}{13}}$$ (16)

The process is repeated until the depth converges to the exact value (difference less than 1cm between two iterations). Then the pressure head *p*(*i*+1) is computed at location *i*+1, at a distance *Δx* upstream, using an explicit Euler finite difference scheme for Eq 2:

$$p\left(i+1\right)=p\left(i\right)-\Delta x\frac{n^{2}Q^{2}{\left(2\left(w+zd\left(i\right)+d\left(i\right)\sqrt{1+z^{2}}\right)\right)}^{4/3}}{{\left(d\left(i\right)\left(w+zd\left(i\right)\right)\right)}^{10/3}}$$ (17)

Once p(*i*+1) is known, the new value d(*i*+1) is computed from Eqs 15 and 16, and so on for the entire length of the channel *L*. The calculation starts at the snout, where we impose a hydrostatic pressure equal to the atmospheric pressure (*p* = 0). We use the longitudinal distribution of glacier ice thickness reconstructed by [34] from glacier deposits (trilogy sequence) in the Eastern part of the Wright Valley, close to the Labyrinth (Fig 2b). Note that the average overburden pressure is 250m, in accordance with the minimum upper-elevation limit (Asgard till) associated with thick ice over the Labyrinth during mid-Miocene time [25]. After calculating the water depth and pressure head along the channel length, the erosion parameters are computed. These values are substituted in Eq 11 to find the erosion rate along the entire channel length. The channel bottom elevation is then modified as a function of the erosion rate, and the computation solution of Eqs 15, 16 and 17 repeated for the new time interval. Channel junctions are modelled by adding the water discharge and sediment load of the tributary channel to the main channel at the junction location.

### 3.4 Morphological analysis of the Labyrinth

We conduct a morphological analysis of the Labyrinth in order to compare model results to real, large-scale landforms. A LiDAR survey was flown in December 2001 yielding elevation measurements with a vertical precision of 0.2m and an average spatial density of 0.23 points per m^2^. A Digital Elevation Model (DEM) is extracted from the LiDAR data by linearly interpolating the elevation data on a Cartesian grid with 2m horizontal resolution (Fig 1).

Two bedrock channels (channel A-B and channel C-D in Fig 1) are analyzed taking more than 200 cross-sections and mapping the longitudinal profiles in ArcGIS. In order to compute channel depth and channel width at each cross section, we define the top of the channel bank as the point with maximum curvature, and the bankfull elevation as the lowest point between the right and left bank elevations. Channel depth is then determined as the difference between the bankfull elevation and the elevation of the lowest point within the cross-section. To determine whether the entire channel bottom profile is concave or convex, we interpolate the bottom elevations with a second order polynomial (parabola) and extract the second derivative. A positive second derivative indicates a concave up profile. Finally, we determine the bottom elevation gradients before and after each junction by linear interpolation of five consecutive bottom elevations.

We use previous work in the Labyrinth to constrain the geometry and flow regime in the simulations. A maximum discharge Q = 40000 m^3^/s yields a velocity of around 10m/s, in the same range of velocities (11–15 m/s) estimated by [21] from field observations in the Labyrinth. Also the size of the clasts between 5 and 80cm agrees well with the data of [21].

## 4. Results

**Fig 4:**
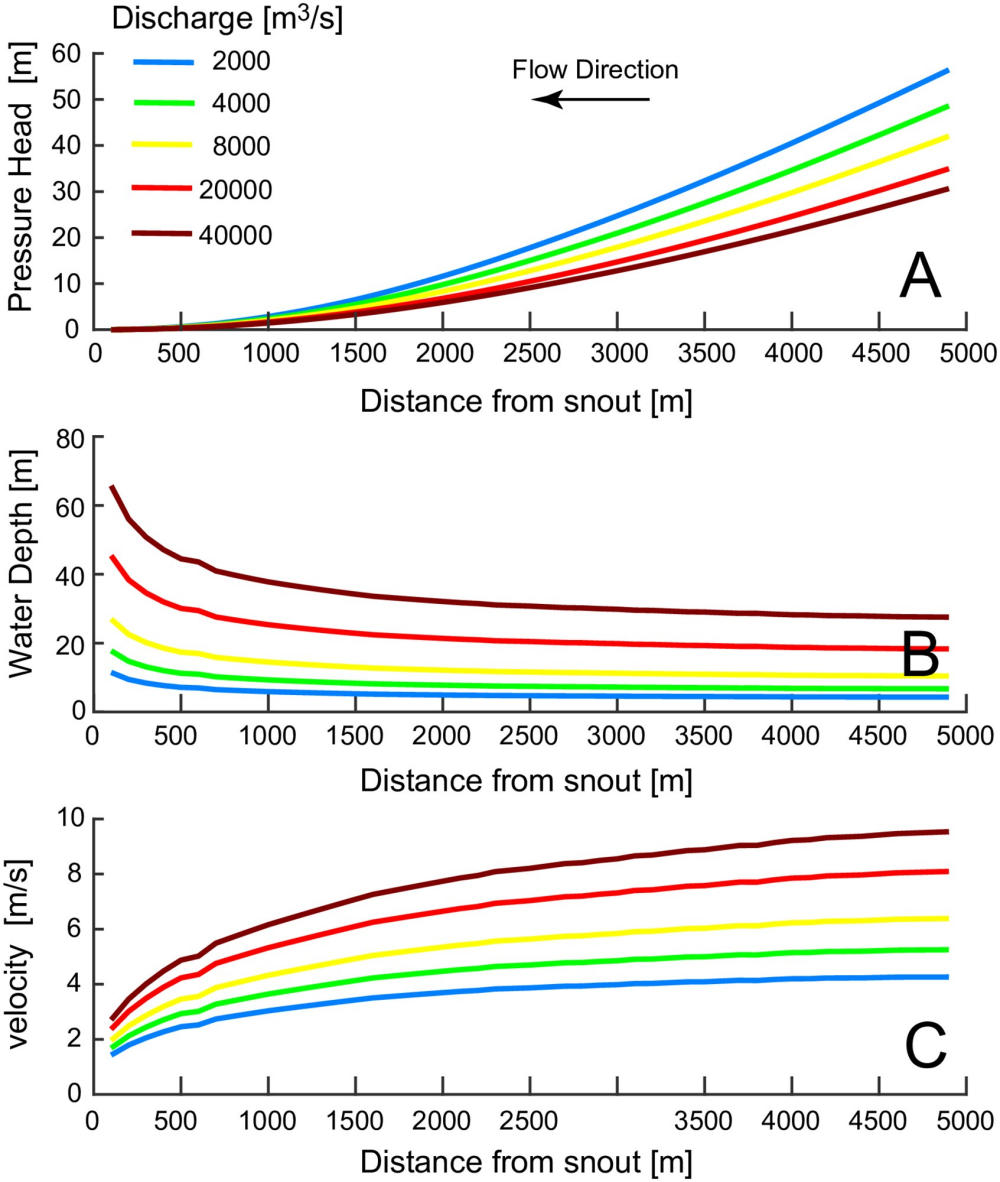
A) Water pressure, B) channel depth, and C) velocity along a subglacial channel for a discharge ranging from 2000 to 40000 m^3^/s. Distances are measured increasing upstream of the glacier’s snout. Different color lines represent different discharges.

The model presented above is used to determine the influence of grain diameter, D_s_, discharge, Q, and sediment flux, q_s_ on the erosion rate of the channel bottom. Other parameters used are based on observations and measurements in the Labyrinth and are listed in Table 1. To study the effect of a single variable, two are set to constant values while the third is allowed to vary within reasonable limits. The distribution of pressure head, water depth, and water velocity along the entire channel length is shown in Fig 4, with Q ranging from 2000 to 40000 m^3^/s. The pressure head profile is concave, with a smaller slope toward the glacier snout (see Fig 4A). This is due to the increasing overburden pressure moving upstream of the snout (Fig 2B). As a result, the velocity of the flow, which is proportional to the square-root of the pressure head gradient, is lower near the snout (Fig 4C). For continuity, the water depth must be higher where the velocity is lower (Fig 4B) so that the water discharge remains constant.

**Fig 5:**
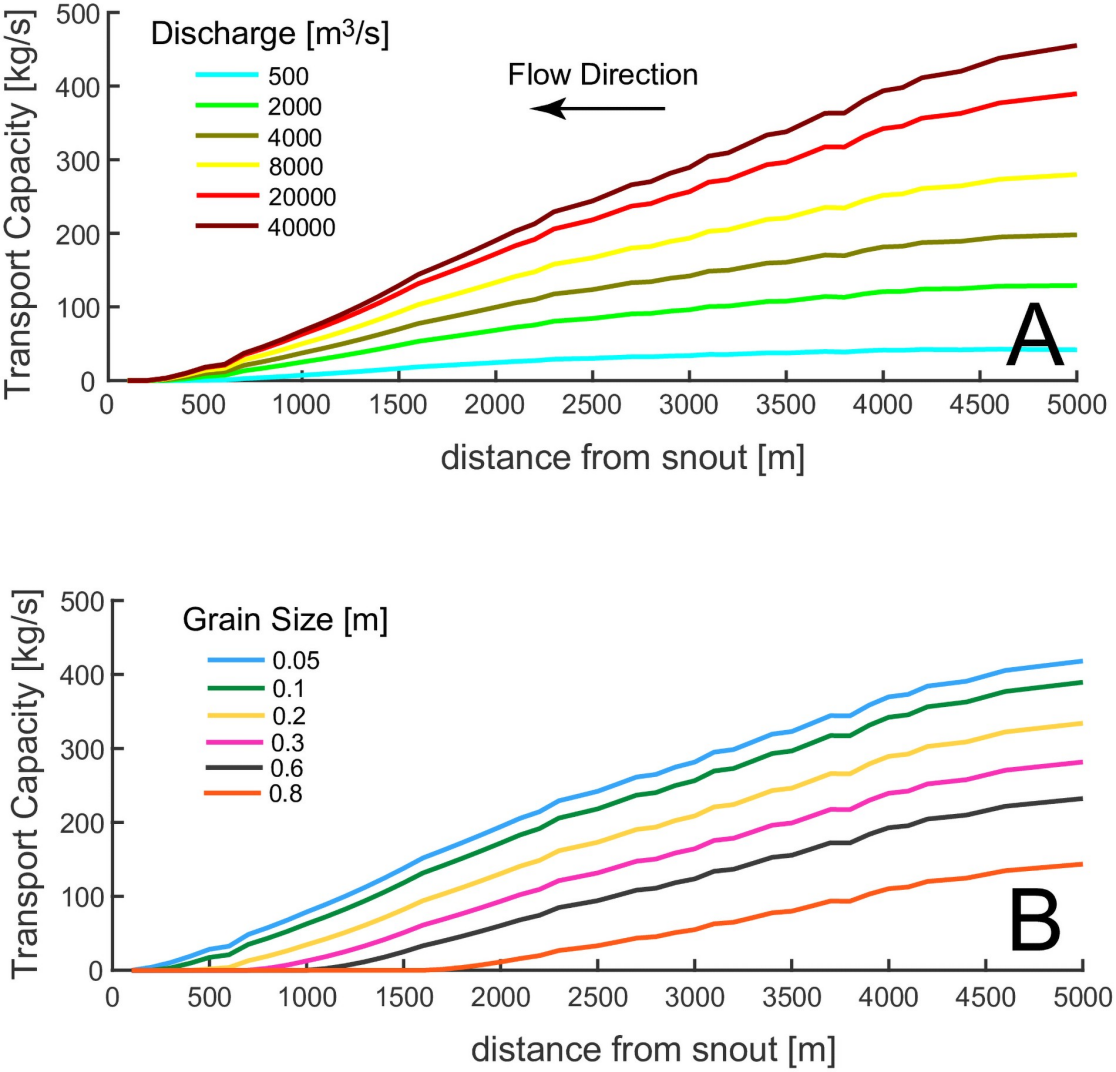
A) Sediment transport capacity along channel length for various water discharges. D_s_ = 10 cm and q_s_ = 40 kg/m/s; B) Sediment transport capacity for various grain size diameters. Q = 20000 m^3^/s, q_s_ = 40 kg/m/s.

As expected, there is a wide range in the pressure head distribution along the channel length, with pressures going to zero (atmospheric) at the snout to 50m. However, despite the fact that the discharge varies by a factor of 20 between the high and low flow cases, the water velocities only triple with the maximum discharge. This important result is caused by a positive feedback between discharge and melting. A large flow dissipates more energy by friction, which tends to increase the water depth by melting the ice roof, thus increasing the channel cross section, reducing the constriction of the flow and consequent increases in velocity at greater discharges. The channel depths, too, have a small range of values, due to the fact that d α Q^9/13^ (Eq 15). Fig 5A shows the variation in the sediment transport capacity, q_t_, along the channel length for different discharges. Higher values of the total discharge yield higher values for the sediment transport capacity because q_t_ α Q^3^ (Eq 13). The transport capacity in general mimics the velocity distribution along the channel (Fig 4C).

### 4.1 Effect of discharge on erosion rate

**Fig 6:**
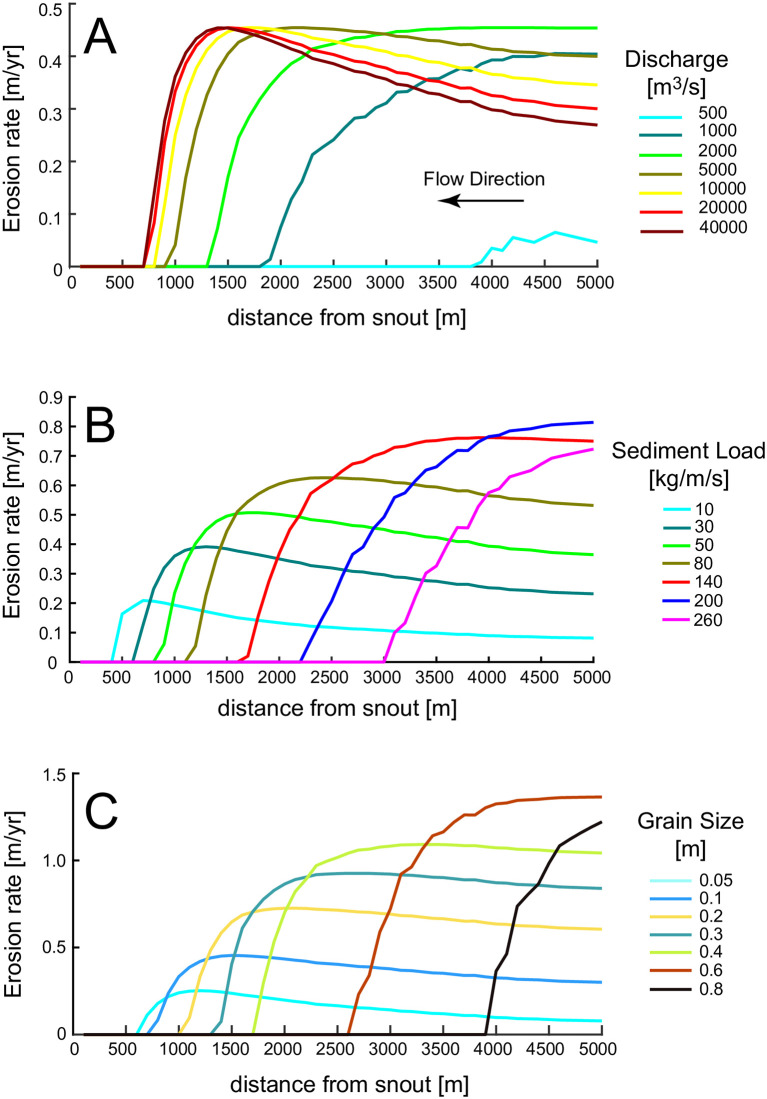
(A) Erosion rate along channel length for different water discharges. D_s_ = 10 cm and q_s_ = 40 kg/m/s. (B) Erosion rate along channel length for different sediment supplies per unit width. Q = 20000 m^3^/s, D_s_ = 10 cm (C) Erosion rate for different grain size diameter of the sediments. Q = 20000 m^3^/s, q_s_ = 40 kg/m/s.

The erosion rate along the channel displays the following: first, erosion reaches a maximum value at a distance from the snout, at which point maximum conditions for bedrock abrasion occur (Fig 6A). For higher velocities, the sediments are mostly in suspension, and therefore they rarely impact the channel bottom. This can also be seen by the transport capacity that dramatically increases upstream of the snout (Fig 5A), limiting the number of tools to abrade the channel bed. Near the snout, where velocities are low, saltation does not occur and abrasion is minimal. Second, the length of channel along which the erosion can occur (*E* > 0) increases with increasing *Q*. This is because larger discharges increase flow velocity and bottom shear stress so that the critical shear stress for erosion is also reached at locations near the snout (Fig 6A). Third, increasing *Q* does not change the peak erosion rate of E ~ 0.45 m/yr. This is because the sediment flux and sediment diameter are fixed in the simulation, and therefore once the discharge is sufficient to mobilize the maximum amount of sediment, the maximum erosion rate is achieved. Addition of more discharge is excessive and does not contribute to bedrock erosion. For low discharges, the peak in erosion is not reached because the velocity is too low.

### 4.2 Effect of sediment flux on erosion rate

**Fig 7:**
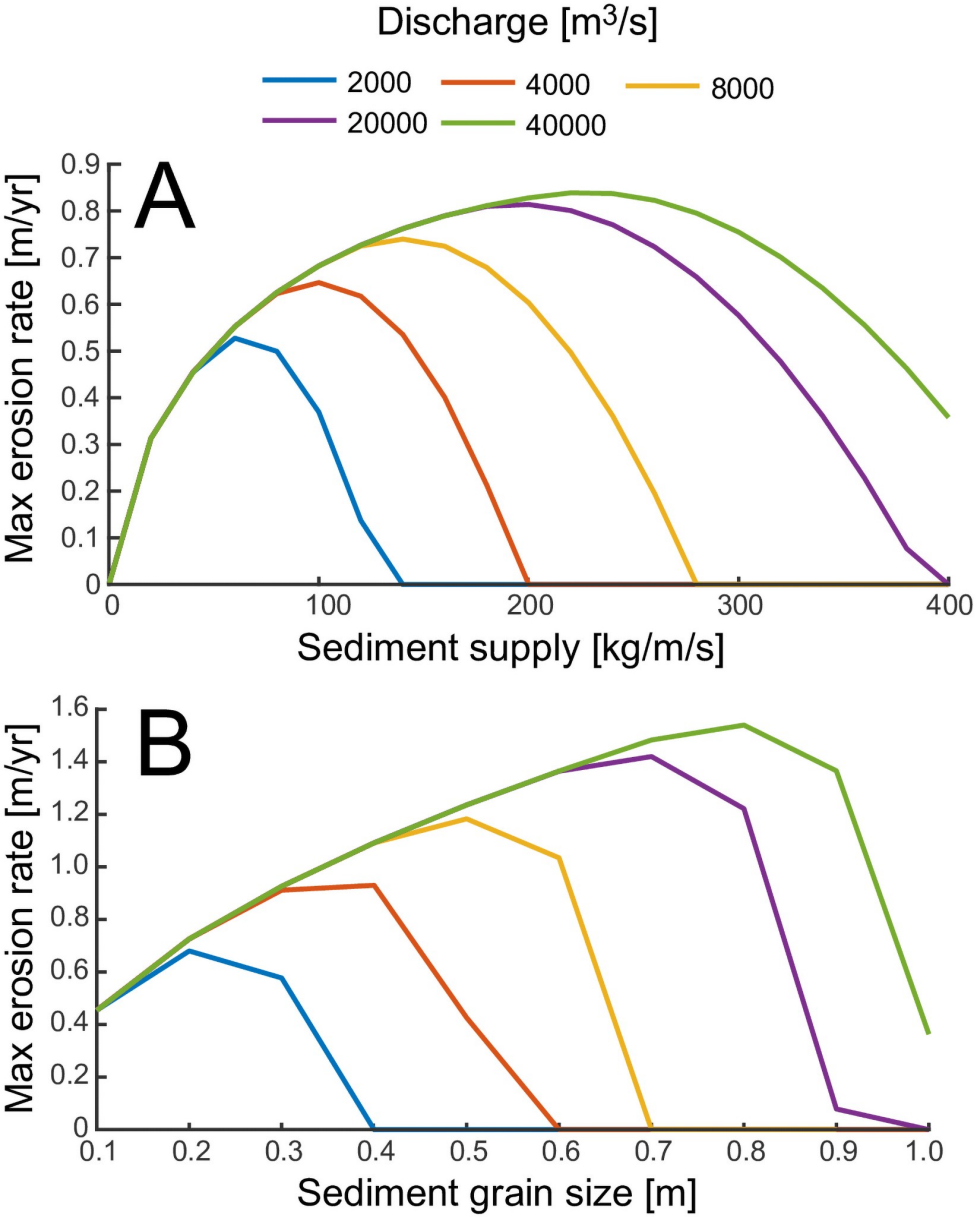
A) Maximum erosion rate along the channel as a function of sediment supply per unit width and water discharge. D_s_ = 10 cm. B) Maximum erosion rate along the channel as a function of sediment grain size and water discharge. q_s_ = 40 kg/m/s.

Several notable results appear in Fig 6B, which shows the erosion rate along the channel for different sediment loads. The maximum erosion rate increases with sediment load below 200 kg/m/s. However, if the sediment load is too high (>200 kg/m/s), most of the channel does not experience erosion, maximum erosion rate occurs at the end of the channel and it is low. This effect can be seen more clearly in Fig 7A, where we report the peak erosion rate as a function of different sediment fluxes and water discharges. The peak erosion rate increases and then decreases with sediment supply. The tools and cover effect, described in [17], explains this behavior. The tools and cover effect also explains why, with increasing sediment load, erosion occurs farther upstream of the glacier snout (see Fig 6B). If the mean flow velocity is too low, the excess sediment covers the channel bed, preventing erosion. Thus, with increasing sediment flux, higher velocities are needed to transport sediment so that erosion of the channel bed can occur. Higher water discharges are able to transport larger sediment loads and increase erosion near the snout (Fig 5a) increasing the maximum erosion along the channel (Fig 7A).

The sediment load might also vary along the glacier bottom. With more material entrained during the erosion of the subglacial channel, the load could become higher close to the snout. This increase in sediment load due to entrainment at the bottom would further accentuate the peak in erosion rate reported in Fig 5A with respect the erosion rate occurring far upstream of the snout.

### 4.3 Effect of sediment grain diameter on erosion rate

In Fig 5B, the sediment transport capacity along the channel length is plotted for different grain diameters with Q = 20000 m^3^/s. Despite the range of grain sizes spanning more than one order of magnitude, the sediment transport capacity remains within a narrow range of values, showing that grain size has limited influence on the sediment transport capacity (Eq 13).

The effect of sediment grain size on maximum erosion rate along the channel is shown in Fig 7B. Note that large diameters lead to high peak erosion rates, since coarser grains impact the bedrock with more energy. However, above a certain grain size, the flow cannot mobilize the sediment, and the maximum erosion quickly drops to zero. On the other hand, the range of velocities over which erosion can take place decreases with grain size, concentrating the erosion upstream of the snout for larger grain sizes (Fig 6C). In fact, as the particle sizes increase, larger mean flow velocities are needed to transport them. Because the mean flow velocities increase with increasing distance from the glacier snout, erosion will shut down (*E* = 0) near the snout.

### 4.4 Morphological evolution of a bedrock channel

**Fig 8:**
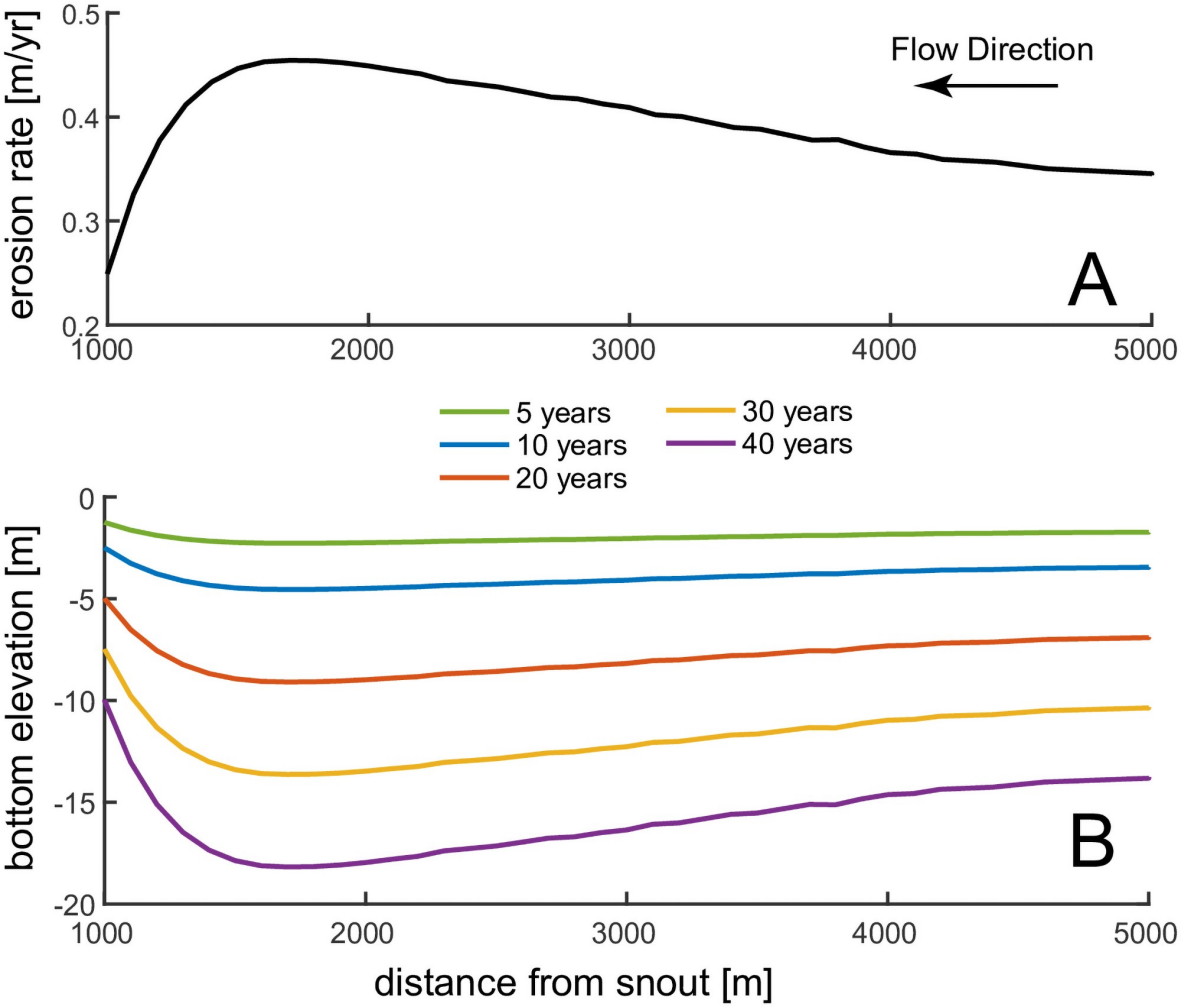
Evolution of a subglacial channel in time: A) erosion rate; B) bottom elevation. At the beginning of the simulations the channel has constant width (100m) and constant bottom elevation. Q = 20000 m^3^/s D_s_ = 10cm q_s_ = 40 kg/m/s.

The model is used to study the evolution of a single channel in time (Fig 8). We start with a straight channel having a constant width of 100m and a fixed discharge of 2000 m^3^/s. Erosion occurs along the entire channel, but is at a maximum 1500m from the snout (Fig 8A). As a result, the entire channel deepens, with the lowest bottom elevation around 700 m upstream of the snout. Erosion rates along the channel do not change in time since they are driven by the pressure head and bottom variations are small. The final channel after 40 years of longitudinal profile evolution has a concave bottom profile, since during the evolution the channel experienced maximum erosion rates at a finite distance from the snout (Fig 8B). Therefore, the non-monotonic relationship between erosion and channel flow has specific geomorphological consequences for channel development, yielding negative bottom gradients near the snout (negative here is defined as upstream).

### 4.5 Channel junctions

**Fig 9:**
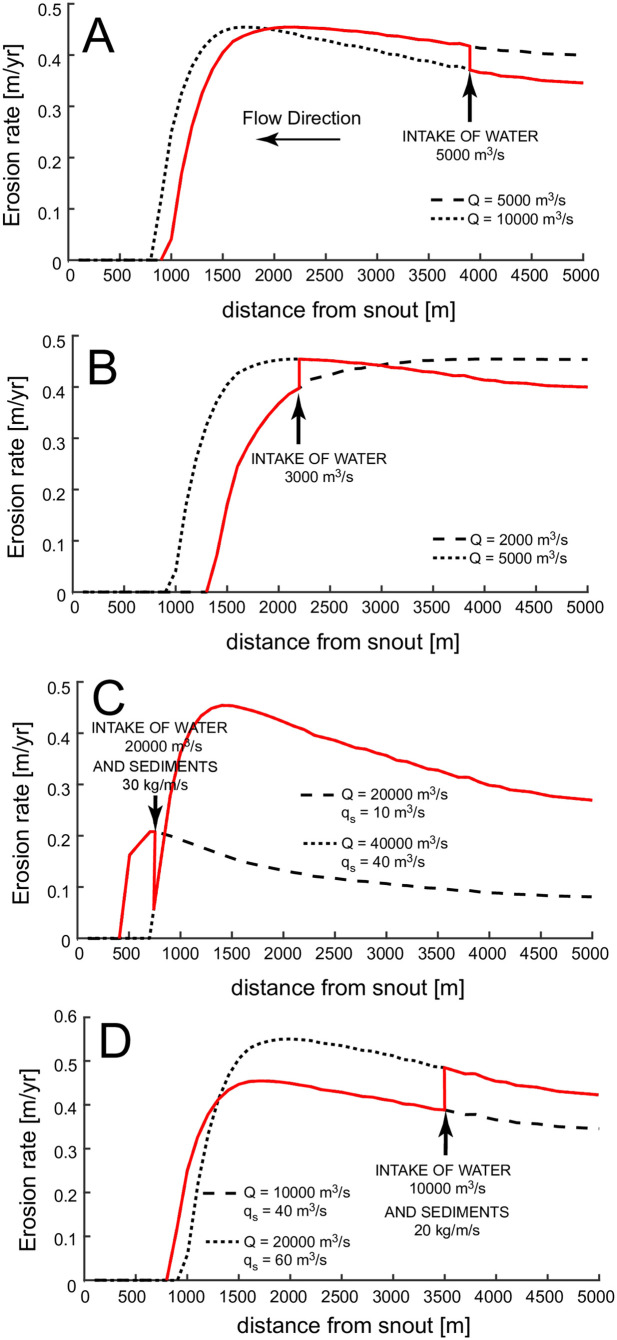
Distribution of erosion rates in a subglacial channel before and after a confluence with another channel. The dashed line is the erosion rate with the initial discharge and sediment load of the main channel, the black arrow is the location where the secondary channel discharges water and sediments, dotted line is the erosion rate with the final sediment discharge and sediment load (sum of the contribution of the two channels), the red line is the distribution of erosion rate before and after the confluence. In A) and B) the secondary channel is adding only water in the main channel while in C) and D) it is also adding sediment load. In A) and C) the erosion rate is higher after the confluence while in B) and D) it is lower.

The effect of a confluence between two channels on the erosion rate is even more striking (Fig 9). The discharge and sediment load of a tributary channel are added at different points along the main channel (black arrows in Fig 9) and the new erosion rate is computed (red lines in Fig 9). The increase in discharge and sediment load in some cases reduces the erosion rate after a junction, leading to a negative bottom gradient. In fact, from Eq 13 we can deduce that q_t_ α Q^3^, so that when two channels with the same discharges and sediment loads merge, they form a new channel with double the sediment load but a much higher transport capacity (i.e. eight times the original value). Since q_s_/q_t_ decreases considerably, so does the erosion rate (Eq 11), with fewer sediment grains available for abrasion at the bottom and most of the transport occurring in suspension.

Fig 9 shows four examples of confluences between two subglacial channels. In the first two we consider an intake of only water (Fig 9A and 9B). If only the discharge increases, the erosion curve of Fig 5A has higher values near the snout, due to increased abrasion near the snout and decreased abrasion in the upstream. The erosion rate of the merged channel depends then on the position of the confluence: if the confluence is far from the snout the erosion rate will decrease after merging, otherwise it will increase (Fig 9A and 9B). The system response is more complex if both channels carry sediments. An increase in sediments supply will increase the peak erosion rate, but move the area of active abrasion upstream of the snout (Fig 6B). Again both configurations are possible, with reduced or enhanced erosion after the junction (Fig 9C and 9D). However, now it is more difficult to determine the final outcome as a function of the confluence position within the channel. From a morphological viewpoint, a decrease in erosion is likely to result in negative bottom gradients after the confluence, while an increase in erosion will tend to deepen the channel. In conclusion, under some conditions, we can expect an increase in bottom elevation after the merging of two channels despite the increase in discharge and sediment load.

### 4.6 Comparison with the geometry of the Labyrinth channels

**Fig 10:**
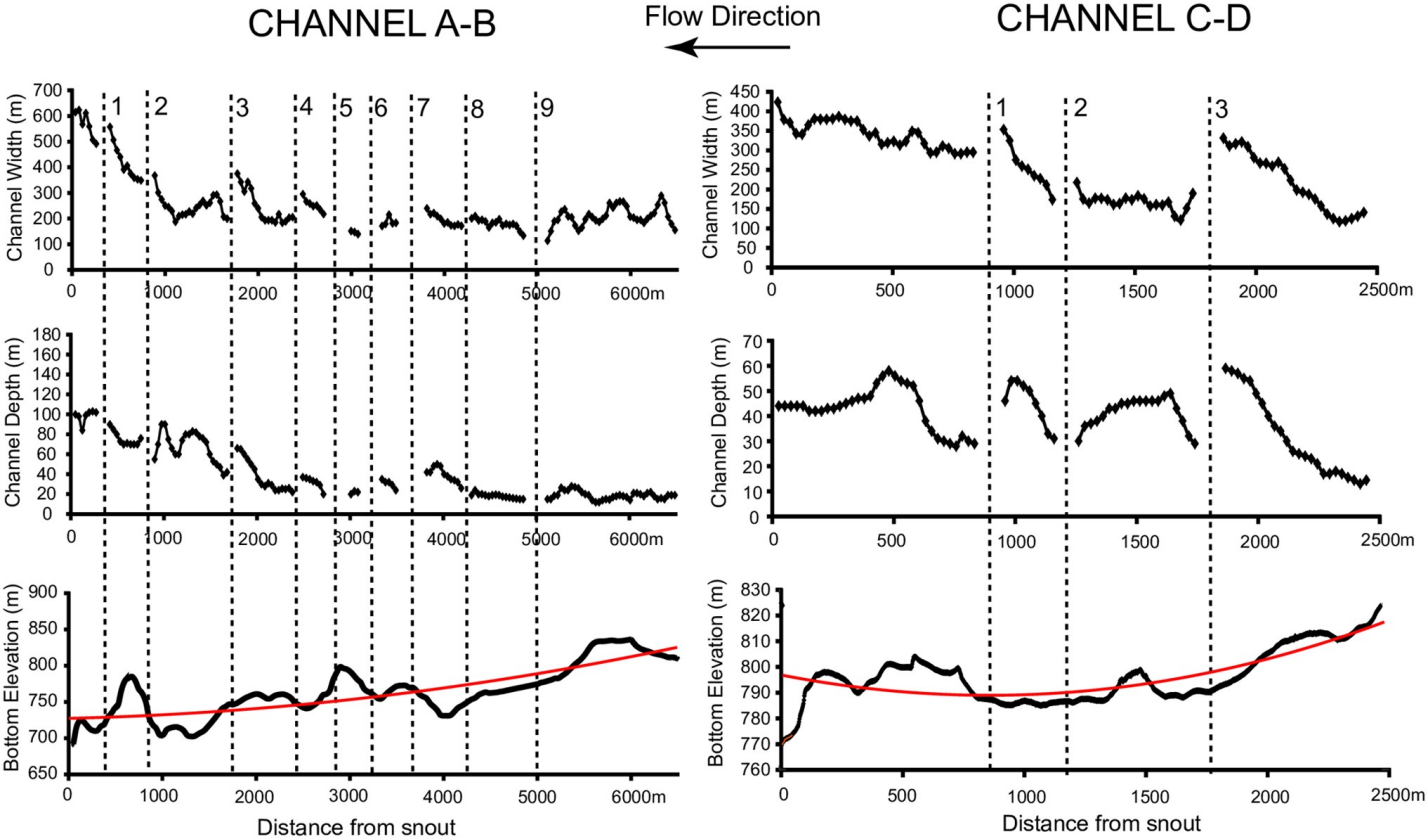
Depth, bottom elevation, and bottom gradient of the two subglacial channels indicated in Fig 1 (channel A-B and channel C-D). The numbers refer to lateral tributaries discharging in the main trunk (see Fig 1). The red lines are a second order polynomial interpolation of the bottom elevation (parabola).

The results of the numerical model are compared to the geometry of the channels in the Labyrinth, Antarctica. The average channel width in the Labyrinth is 150 m and the average depth is 50 m (Fig 10). These averages are most likely overestimates of the actual values because as the channels decrease in size, it becomes increasingly difficult to find long channel reaches not interrupted by junctions, so that larger channels were preferentially analyzed. Tributary channels discharging in the main trunk are indicated with numbers in Fig 10 (see Fig 1 for the planimetric location of the junctions). At each junction, the width and depth of the main channel is undefined since the lateral channel banks are not present; this result in a gap in the data of Fig 10. The width of channel A-B is relatively constant for more than 5 km upstream, justifying the assumption of constant base width in the model (Fig 10). The width tends to increase only at the terminus, growing from 200m to 600m in the last kilometer. The width of channel C-D is more variable, and grows from 250m to 350m near the terminus. Channel C-D is smaller, and might have experienced larger variations of discharge because of tributaries, possibly affecting channel widths. Channel depths do not increase to maintain the same width-to-depth ratio, but rather fluctuate or increase by only a few tens of meters near the channel terminus: the depth of channel A-B increases from 70 to 100m, while the depth of channel C-D oscillates between 30 and 50m in the last kilometer of the channel. Only in a few cases channel widening is associated with an increase in channel depth. This is evident in Channel A-B, which aggregates a large number of tributaries (Fig 10). The longitudinal bottom profile is concave up, similarly to the model results of Fig 8B. In fact, the second derivative of the interpolating parabola is positive and equal to 1.87 10^−6^ m^-1^. We therefore ascribe the concave profile to a reduction of erosion rate within the first hundreds of meters upstream of the snout produced by a decrease in transport capacity. The bottom elevation profile of channel C-D is even more concave up, with a second derivative of the interpolating parabola equal to 1.08 10^−5^ m^-1^.

**Fig 11:**
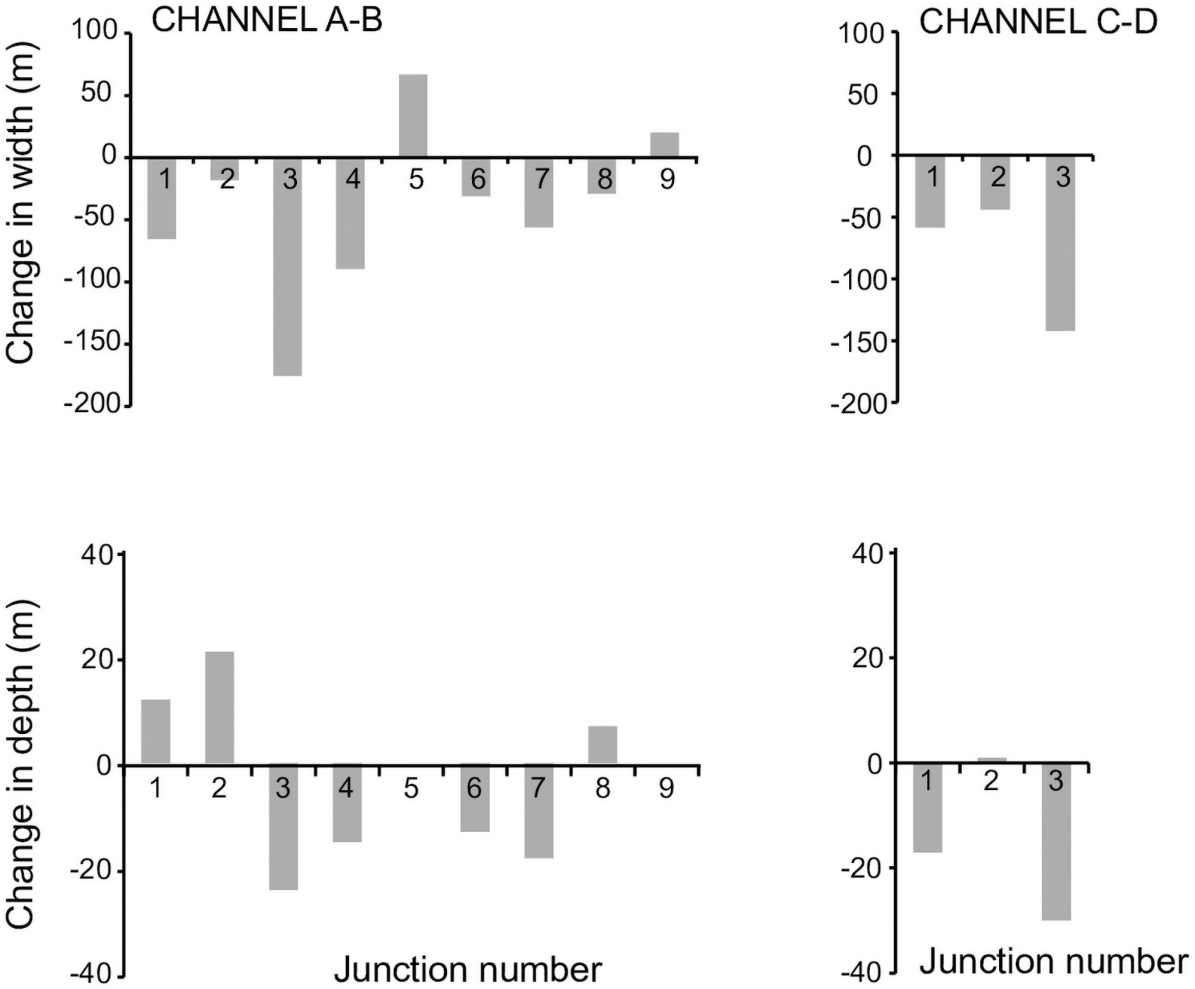
Change in channel width and depth after a junction for channels A-B and C-D in Fig 1. Positive values mean an increase in width and depth. The numbers refer to lateral tributaries discharging in the main trunk (see Fig 1).

**Fig 12:**
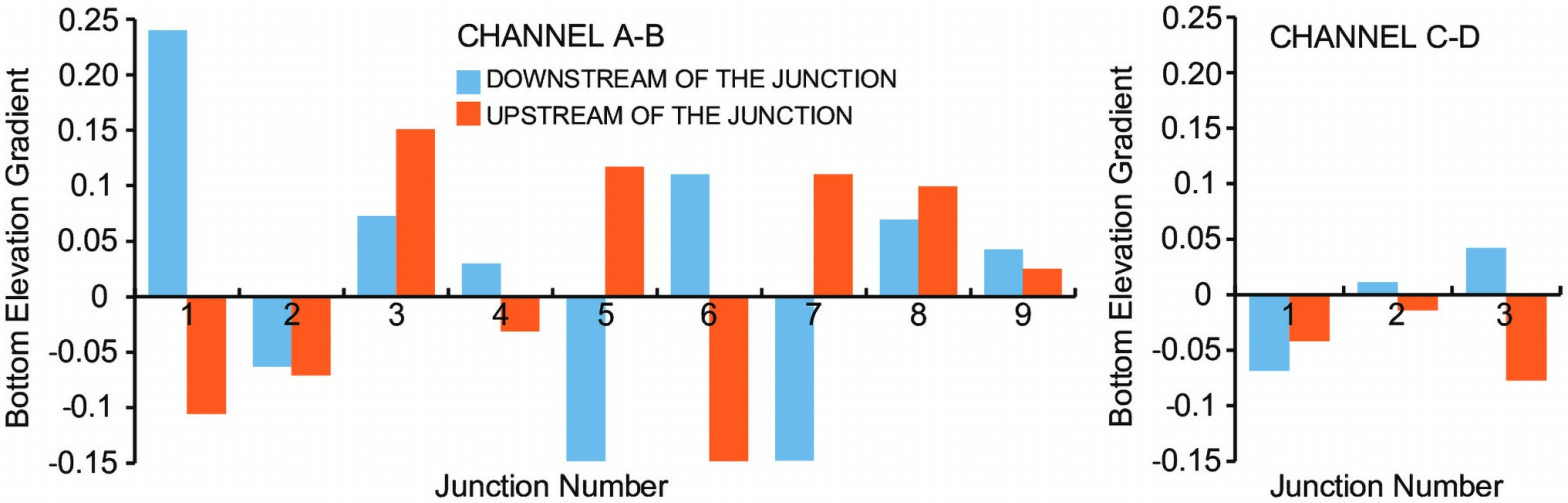
Bottom elevation gradient before and after each junction for channels A-B and C-D in Fig 1. The numbers refer to lateral tributaries discharging in the main trunk. Negative is defined as upstream.

At tributary junctions, channels show different behaviors. In Fig 11 we plot the difference between the width upstream and downstream of the junction and the channel depth upstream and downstream of the junction (positive values indicate upstream enlargement and deepening). In some cases, the cross section becomes larger upstream of the junction; in other cases, it becomes smaller. In many channels the depth diminishes upstream of a junction (channel A-B junction, 3,4,6,7 channel C-D junction 1,3), while in only three junctions the depth becomes noticeably larger (channel A-B junction 1,2,8). Several channels display negative bottom elevation gradients upstream of the junction (channel A-B junction 1,2,4,6 channel C-D junction 1,2,3, Fig 12), but only few experience a switch from positive to negative gradient at the junction, with the bottom elevation decreasing downstream of the junction and increasing upstream (channel A-B junction 1,6, Fig 12). In all the others, the bottom elevation starts to decrease after a few hundred meters downstream of the junction (channel A-B junctions 3,5,8).

The fact that a reverse gradient develops upstream of a junction might be due to local hydrodynamic processes that cannot be captured by our simplified model. For example, the three dimensional turbulent flow at the confluence can lead to scour holes [35, 36], as observed in the Labyrinth [21].

We interpret an increase in bottom elevation as a decrease in erosion power of the subglacial flow, triggered by the confluence. Our model results indeed show that an increase in discharge enhances transport capacity, thus maintaining most of the sediment in suspension and limiting grain impacts at the bottom and therefore abrasion. If sediment load also increases upstream of the junction, the trend can be reversed: more sediment provides tools for abrasion that counteract the increase in transport capacity, as indicated by our model simulations. An increase in bottom elevation can also be caused by channel widening if the discharge is constant. However, the discharge is increasing downstream of a confluence, and the channel width is decreasing downstream of most confluences analyzed here (Fig 11a).

## 5. Discussion

The results of the numerical model show that the erosion rate does not increase monotonically downstream along the length of a subglacial channel. The location of maximum erosion rate depends on discharge, sediment flux, and grain size (Fig 6). The peak in erosion rate is closer to the glacier’s terminus for increasing discharge, but it moves upstream for increasing sediment fluxes and grain diameters.

### 5.1 Implications for the formation and morphology of subglacial channels

Here we have applied the balance between channel ice melting and ice creep developed for channels cut into ice (R-channels) to channels incised into bedrock (N-channels). Our results indicate that bedrock abrasion can be fast, in the order of decimeters per year. This fast erosion rate can outpace basal glacial erosion, carving large channels into bedrock beneath glaciers. Fast bedrock erosion leads to channel deepening, and a low width-to-depth ratio, as observed in the channels of the Labirinth [21, 24]. Fast bedrock erosion and the formation of N-channels would allow the evacuation of melting water, reducing basal pressure and favoring the formation of few large channels rather than a diffuse drainage system of small cavities [12]. Reverse gradients of bottom elevations, particularly after junctions, were already observed by other researchers [22, 23], and utilized as a proof of pressurized flow. However, previous studies did not indicate how these negative channel gradients might have developed. Here for the first time we show that the formation of negative gradients in subglacial bedrock channels can be ascribed to the non-monotonic erosion rate along the channel triggered by the tool and cover effect.

### 5.2 Implications for the estimation of formative discharges

[24] modeled continuous, steady state subglacial fluxes beneath Pine Island and Thwaites glaciers in Antarctica. The highest calculated discharge in a channel was 140 m^3^/s, which is clearly too low to carve channels larger than those found in the Labyrinth. Based on these simulations [24], concluded that the channels offshore of present-day Pine Island and Thwaites glaciers were carved by episodic, large-scale events, like outburst floods.

Our results confirm that the discharge needed to erode the bedrock channels of the Labyrinth is much larger than the continuous, steady state subglacial fluxes estimated by [24] (see Fig 6a). Therefore, also the Labyrinth channels must have been scoured by outbursts of water and sediment fluxes triggered by the drainage of subglacial lakes. However, the discharge responsible for the carving of these channels can be smaller than the maximum values estimated by [21]. First, the channels do not need to be at bankfull to erode the channel bottom, water depths of few meters are enough to develop the water velocities required for bedrock erosion (Fig 4). Second, peak erosion can be reached for velocities much lower than the 11–15 m/s estimated by [21]. In Fig 6a a discharge 20 times lower than the discharge triggering a 10 m/s velocity results in the same rate of bedrock erosion. Finally, the erosion rates computed by our model do not exceed 2 m/yr. This indicates that the channels formed in years, and not during an event that lasted few days.

### 5.3 Alternative erosive processes

Here we assume that the only erosive process in a subglacial bedrock channel is abrasion by saltating particles at the channel bed. However, both plucking and cavitation can play an important role in bedrock erosion [21, 37, 38]. Hydraulic plucking is favored by closely-spaced bedrock jointing as present in the Labyrinth [21, 37, 39], while cavitation may occur due to the high water velocities.

The cavitation inception index is defined as [37]:

$$\sigma =\frac{p-p_{v}}{\frac{1}{2}\rho_{w}v^{2}}$$ (18)

where *p*_*v*_ is the vapor pressure. Cavitation is present when the index is below one. Values of hydrostatic pressure and velocity computed by our model in subglacial channels (see Fig 4) indicate that the index is less than one for hydrostatic pressures below 5m, which only occurs for a limited distance near the snout. Therefore, most of the channel is not subject to cavitation due to the large hydrostatic pressure.

Accounting for plucking would most likely increase the erosion rate in the channel (hydraulically plucking a dolerite boulder requires a lot less time than eroding it by abrasion). However, erosion by plucking is a monotonic function of water velocity [38] and water pressure [40], so this process cannot yield concave bottom profiles or a reduction in bottom elevation after junctions.

### 5.4 Channel incision over time

As noted in Fig 6, to determine the timescale of channel formation, it is important to determine sediment load and grain size distribution, since the peak erosion rate along a subglacial channel is independent of discharge. Moreover Fig 6A implies that there are two different styles of channel erosion and related evolution. At low discharge the erosion is concentrated upstream of the snout. For large discharges the erosion propagates toward the snout and is distributed along most of the channel. In theory it would then be possible to extract information on the past flow regimes from the planimetric morphology of the channel, assuming a stationary ice front. A constant discharge would form a channel with either pronounced erosion upstream of the snout (low discharge) or in a channel’s middle reaches (large discharge). A variable discharge, as during a meltwater pulse, would instead result in a flatter channel, since different discharges would preferentially erode different channel locations, thus moving the peak erosion rate back and forth along the channel length.

### 5.5 Limitations of the model

Here we have focused on the erosion of the channel bottom only, when in reality the entire channel cross section might evolve due to abrasion [41]. However, more sophisticated models that track the evolution of a channel cross section by computing the distribution of bottom shear stresses show that, for a given grain size, the width-to-depth ratio stays relatively constant during channel evolution (e.g. [42]). If channel widening is somehow proportional to deepening, the results presented herein are still qualitatively valid.

A wider channel reduces the water pressure, and therefore flow velocity and water depth. A slow flow would shift erosion upstream of the snout (see Fig 6A), since the velocity is too low to transport sediments at the snout (Fig 4C). Only the part of the channel far from the snout would then enlarge, but always with a peak erosion rate at a certain distance from the snout, since far upstream of the snout the transport capacity is so high that most sediment is transported in suspension.

Our model can be applied to a large range of water discharges and sediment loads, encompassing both continuous, steady-state flow condition and episodic outbursts triggered by volcanic activity and drainage of subglacial lakes. Its flexibility will allow its application to similar systems in Antarctica, as, for example, the subglacial channels beneath the Pine Island and Thwaites glaciers, West Antarctica [24]. Future research will extend the present model to the entire channel cross section, thus determining the relationship between bedrock erosion, channel depth and channel width, as has been done in recent years for bedrock channels (e.g. [43]).

## 6. Conclusions

A numerical model is developed combining the model of subglacial channels proposed by [1] and theory of bedrock abrasion put forward by [17]. Model results lead to the following key conclusions:

1. Bedrock abrasion in subglacial channels peaks at an intermediate distance from the snout for a wide range of sediment grain sizes and sediment loads. Close to the snout the flow velocity is low and the sediment particles cannot be mobilized. Far from the snout the velocity is too high and a large fraction of the sediments is transported in suspension, thus limiting abrasion by particle impacts at the bottom.
2. This particular distribution of erosion along the channel yields a concave profile, which is typical of the subglacial bedrock channels of the Labyrinth, Antarctica.
3. At channel junctions the complex feedbacks among transport rate, sediment supply, and transport capacity can lead to a decrease in erosion rate after the junction under the right set of conditions. We ascribe the reduction of channel depth at several channel junctions in the Labyrinth, Antarctica to this decrease in bedrock abrasion.
